# In *Candida glabrata*, ERMES Component *GEM1* Controls Mitochondrial Morphology, mtROS, and Drug Efflux Pump Expression, Resulting in Azole Susceptibility

**DOI:** 10.3390/jof9020240

**Published:** 2023-02-10

**Authors:** Michiyo Okamoto, Keiko Nakano, Azusa Takahashi-Nakaguchi, Kaname Sasamoto, Masashi Yamaguchi, Miguel Cacho Teixeira, Hiroji Chibana

**Affiliations:** 1 Medical Mycology Research Center, Chiba University, Chiba 260-8673, Japan; 2 iBB—Institute for Bioengineering and Biosciences, Instituto Superior Técnico, Universidade de Lisboa, 1049-001 Lisbon, Portugal; 3 Associate Laboratory i4HB—Institute for Health and Bioeconomy at Instituto Superior Técnico, Universidade de Lisboa, 1049-001 Lisbon, Portugal

**Keywords:** ERMES component, *GEM1*, mitochondrial dynamics, mitochondrial fission, *PDR1*, *CDR1*, *CDR2*

## Abstract

Mitochondrial dysfunction or morphological abnormalities in human pathogenic fungi are known to contribute to azole resistance; however, the underlying molecular mechanisms are unknown. In this study, we investigated the link between mitochondrial morphology and azole resistance in *Candida glabrata*, which is the second most common cause of human candidiasis worldwide. The ER-mitochondrial encounter structure (ERMES) complex is thought to play an important role in the mitochondrial dynamics necessary for mitochondria to maintain their function. Of the five components of the ERMES complex, deletion of *GEM1* increased azole resistance. Gem1 is a GTPase that regulates the ERMES complex activity. Point mutations in *GEM1* GTPase domains were sufficient to confer azole resistance. The cells lacking *GEM1* displayed abnormalities in mitochondrial morphology, increased mtROS levels, and increased expression of azole drug efflux pumps encoded by *CDR1* and *CDR2*. Interestingly, treatment with N-acetylcysteine (NAC), an antioxidant, reduced ROS production and the expression of *CDR1* in Δ*gem1* cells. Altogether, the absence of Gem1 activity caused an increase in mitochondrial ROS concentration, leading to Pdr1-dependent upregulation of the drug efflux pump Cdr1, resulting in azole resistance.

## 1. Introduction

*Candida* species can cause severe systemic infections in immunocompromised patients and are associated with high mortality rates [1,2]. *Candida albicans* is the most common cause of candidiasis. However, the frequent use of azole antifungals has led to the emergence of candidiasis caused by non-*albicans Candida* species, which display lower susceptibility to azoles [3,4]. Among these non-*albicans Candida* species, *Candida glabrata* is the first or second leading cause of candidemia in many countries [5,6,7]. The virulence mechanisms of *C*. *glabrata* are different from those of *C*. *albicans*; the most crucial difference is that *C*. *glabrata* does not cause significant damage to the host cell and does not provoke a strong response by the host immune system [8]. Treatment of *C*. *glabrata* infections is made difficult by the limited knowledge of its pathogenicity and its low susceptibility to azoles. Therefore, a better understanding of the mechanisms underlying azole resistance is critical for the treatment of *C*. *glabrata*.

Azole antifungals selectively inhibit 14α-lanosterol demethylase (Erg11) in ergosterol biosynthesis, leading to a depletion of ergosterol and an accumulation of the toxic sterol dimethylcholesta-8,24(28)-dien-3β,6α-diol [9]. In *C*. *glabrata,* azole resistance in clinical isolates is mainly caused by activating mutations in the transcription factor Pdr1, resulting in the overexpression of multi-drug transporters of the ATP-binding cassette (ABC) family, such as Cdr1 and Cdr2 [6,10,11,12,13]. Upregulation of ABC transporters by Pdr1 has also been reported in mitochondrial dysfunction, leading to azole resistance [11,14]. Mutations in the mitochondrial genome or loss of mitochondria cause the so-called petite phenotype, observed in clinical isolates obtained from patients treated with azoles [15,16] or upon in vitro exposure to high concentrations of azoles [11]. Azole exposure induces a temporary loss of mitochondrial function [17]. However, it is unclear how azoles cause mitochondrial dysfunction and what molecular mechanisms of mitochondrial dysfunction contribute to the azole resistance mediated by ABC transporters. Mitochondrial fission and fusion are crucial for mitochondrial functioning. Mutants with defects in mitochondrial fission have been found to be azole-resistant in *Aspergillus fumigatus* [18], and deletion of *CgSHE9*, which is involved in mitochondrial inner membrane fission, has been reported to confer resistance to fluconazole in *C*. *glabrata* [17]. Therefore, it is important to clarify the mechanism through which mitochondrial dysfunction caused by defects in mitochondrial fission leads to azole resistance.

**Figure 1:**
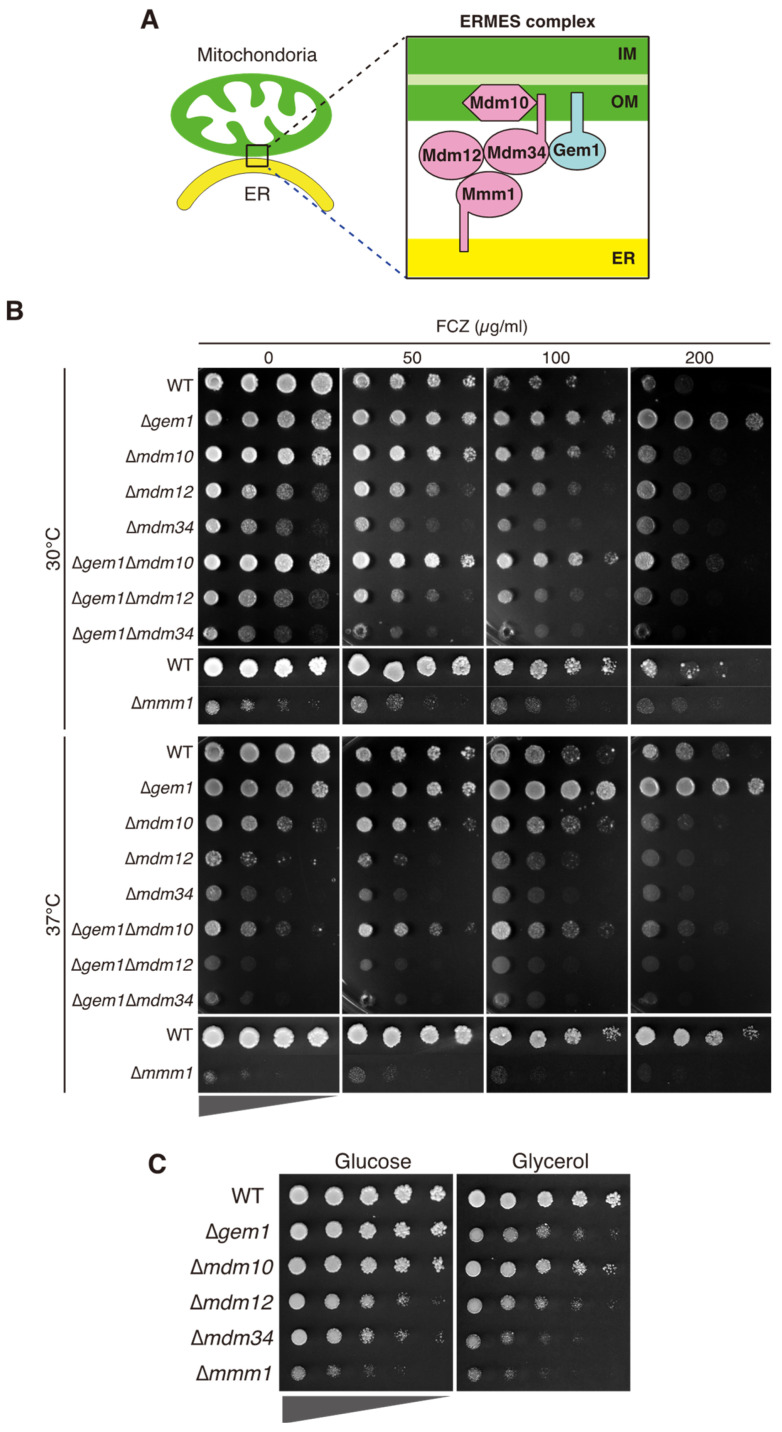
Resistance to azoles by deletion of *GEM1*. (**A**) Schematic diagram of the ERMES complex, based on a report in *S*. *cerevisiae*. OM, outer membrane. IM, inner membrane. (**B**) Growth of strains lacking ERMES components (Δ*mdm12*, Δ*mdm34*, Δ*mdm10*, Δ*mmm1*, and Δ*gem1*) in the presence or absence of fluconazole. The cells were diluted to OD_600_ (optical density at 600 nm) of 0.5 in water and spotted in 4-fold serial dilutions (indicated by triangles) on agar plates of minimal SD medium containing the indicated concentration of fluconazole. All cells except for Δ*mmm1* cells were incubated for 2 days (upper panel), and Δ*mmm1* cells were incubated for 3 days at 30 °C or 37 °C. (**C**) Growth of strains lacking ERMES components on the plate containing glycerol as the carbon source. The cells were spotted in 4-fold serial dilutions on the minimum medium containing glucose or 3% glycerol and incubated for 3 days at 30 °C.

It has become clear that different organelles are in physical contact with each other and exchange substances and information through their contact sites [19,20]. In *Saccharomyces cerevisiae*, the outer mitochondrial membrane is connected to the endoplasmic reticulum (ER) through the contact site formed by the ER-mitochondria encounter structure (ERMES) complex [21]. The ERMES complex is composed of four core subunits: an ER-resident protein Mmm1, a cytosolic protein Mdm12, and mitochondrial outer membrane proteins Mdm10 and Mdm34 [21] (Figure 1A). Gem1, a Rho GTPase of the outer mitochondrial membrane, was recently identified as a subunit of the ERMES complex and is suggested to be involved in the regulation of its function [22,23]. Mdm12, Mdm34, and Mmm1 have synaptotagmin-like mitochondrial-lipid-binding protein (SMP) domains [24], and the ERMES complex mediates lipid transport [25,26]. The ERMES complex is also involved in mitochondrial fission, the distribution of mtDNA, and mitophagy [27,28,29,30,31,32,33].

The ERMES complex was found to be involved in *C. albicans* and *A. fumigatus* pathogenicity, suggesting its potential as a target for new antifungal drugs [34,35,36]. Similar to *S. cerevisiae*, inactivation of the ERMES complex results in the disruption of mitochondrial tubular morphology in these pathogens. In *C. albicans,* the ERMES complex is involved in immune system evasion [35] and cell wall stress responses [34]. Recently, the yeast Gem1 homolog was isolated as GemA in *A*. *fumigatus* and was shown to be required for azole susceptibility, hyphal growth, virulence, and cell wall integrity [37].

In the present study, the link between the ERMES complex and azole resistance was investigated in *C. glabrata*. Upon the discovery that *GEM1* deletion leads to azole resistance, its impact on mitochondrial morphology, Reactive Oxygen Species (ROS) accumulation, and the activation of azole drug efflux pumps was evaluated, providing new clues on the mechanisms of azole resistance associated with mitochondrial dysfunction.

## 2. Materials and Methods

### 2.1. Strains and Media

The yeast strains used in the present study are listed in Table S1. Yeast cells were grown in rich medium (YPD; 2% peptone, 1% yeast extract, and 2% glucose) or minimal medium (SD; 0.17% yeast nitrogen base without amino acids and ammonium sulfate, 2% glucose, 5% ammonium sulfate, and appropriate amino acids) at 30 °C or 37 °C.

### 2.2. Strain and Plasmid Construction

Deletion of *GEM1*, *MDM10*, *MDM12*, *MDM34,* or *MMM1* was carried out by homologous recombination with appropriate DNA cassettes, amplified by polymerase chain reaction (PCR) using the pHIS906 plasmid containing *HIS3*, as described previously [38]. The deletion of *MDM10*, *MDM12*, or *MDM34,* in a *Δgem1* background, was performed using the pBV65 plasmid, containing the nourseothricin resistance marker and *loxP* recombination target sites, as reported previously [39]. Strains expressing Mdm34-mCherry or Tom70-GFP were constructed by homologous recombination using PCR-amplified cassettes obtained with the primers listed in Table S2, as previously reported [40]. Plasmids pFA6a-mCherry-natNT2 or pFA6a-GFP(S65T)-His3MX6 were used as templates. The insertion of these cassettes into the transformed cells was verified using colony PCR. To construct pGRB-GFP-GEM1, the promoter region of *GEM1* was amplified by PCR using the GFP-GEM1-F1/GFP-GEM1-R1 primer pair, the sequence encoding GFP using the GFP-GEM1-F2/GFP-GEM1-R2 primer pair, and *GEM1* and its terminator region using the GFP-GEM1-F3/GFP-GEM1-R3 primer pair (Table S2). The obtained DNA fragments containing the overlapping end were fused by PCR and inserted into the *Sac*I/*Kpn*I site of the pGR2.1 plasmid (CEN, *URA3*) [41]. The plasmids pGRB-GFP-gem1(T19A), pGRB-GFP-gem1(E216A), pGRB-GFP-gem1(E344A), or pGRB-GFP-gem1(S452A) were constructed by site-directed mutagenesis of the pGRB-GFP-GEM1 plasmid using the KOD PCR master mix (Toyobo, Osaka, Japan).

### 2.3. Quantitative RT-PCR

Cells were cultivated in SD minimal medium at 37 °C until the exponential phase was reached, collected by centrifugation, and washed twice with sterile distilled water at 4 °C. Total RNA was extracted using ISOGEN (Nippon Gene, Tokyo, Japan), and cDNA was synthesized using ReverTra Ace and random primers (Toyobo, Osaka, Japan). The amount of RNA for *CDR1*, *CDR2*, or *PDR1* was determined by quantitative real-time PCR (qRT-PCR) on a LightCycler^®^ 96 System (Roche Diagnostics, Mannheim, Germany) with SYBR Green detection using the Thunderbird SYBR qPCR mix (Toyobo). The transcription levels were normalized to those of *TEF1*, a housekeeping gene that encodes an elongation factor 1. The following primer pairs were used; PDR1-F/PDR1-R, CDR1-F/CDR1-R, CDR2-F/CDR2-R, and TEF1-F/TEF1-R (Table S2). PCR conditions were as follows: pre-denaturation at 95 °C for 1 min, followed by 40 cycles of denaturation at 95 °C for 15 s, and annealing/extension at 60 °C for 1 min.

### 2.4. Protein Localization by Fluorescence Microscopy 

Δ*gem1* cells expressing a C-terminal mCherry-tagged Mdm34 under its endogenous promoter were transformed with pGRB-GFP-*GEM1*, pGRB-GFP-gem1(T19A), pGRB-GFP-gem1 (E216A), pGRB-GFP-gem1 (E344A) or pGRB-GFP-gem1(S452A). These transformants were cultured until the exponential phase in minimum SD medium lacking uracil at 30 °C and observed using a confocal microscope equipped with a 100× objective lens, Stellaris 5 (Leica Microsystems, Wetzlar, Germany). Images were processed by lightning deconvolution using LAS X software.

### 2.5. Mitochondrial Reactive Oxygen Species Quantification

Mitochondrial Reactive Oxygen Species (mtROS) production was monitored by staining with the MitoTracker Red CM-H2XROS (Thermo Fisher Scientific, Waltham, MA, USA). The cells were incubated with 0.5 µM MitoTracker Red CM-H2XROS for 20 min in the dark, washed twice with fresh medium, and then observed under a BZ-9000 microscope (Keyence, Osaka, Japan) equipped with a 100× oil-immersion objective lens.

## 3. Results 

### 3.1. CgGem1 Is Involved in Azole Susceptibility

In *C*. *glabrata,* the predicted components of the ERMES complex, based on their homology to their *S. cerevisiae* counterparts, are encoded by CAGL0C02695g (*MDM10*), CAGL0E02365g (*MDM12*), CAGL0I07007g (*MDM34*), CAGL0D05698g (*MMM1*), and CAGL0M12276g (*GEM1*). To examine the involvement of the ERMES complex in the susceptibility of *C*. *glabrata* to azole drugs, each ERMES gene was individually deleted, and the resulting strains were tested for fluconazole susceptibility. The deletion of *MDM12*, *MDM34*, or *MMM1* led to growth defects in the SD medium at 30 °C and was more pronounced at 37 °C (Figure 1B). Deletion of *GEM1* or *MDM10* genes also led to a slight reduction in growth compared to the wild-type strains. Notably, Δ*gem1* cells exhibited reduced susceptibility to fluconazole compared to wild-type cells at 30 °C and 37 °C, suggesting that Gem1p is involved in fluconazole-induced growth inhibition. The deletion of the remaining ERMES components resulted in decreased growth by fluconazole, similar to that of wild-type strains. Cells lacking *GEM1*, *MDM12*, or *MDM34* grew more slowly on the minimum. The medium contains the nonfermentable carbon source glycerol, suggesting a mitochondrial dysfunction (Figure 1C). 

To further examine whether fluconazole resistance induced by the deletion of *GEM1* is dependent on other ERMES components, we constructed double deletion mutants of *GEM1* and each of the genes encoding ERMES subunits, and investigated their growth in the presence of fluconazole. Deletion of *MDM10*, *MDM12*, or *MDM34* in Δ*gem1* cells led to the abrogation of the azole resistance phenotype observed in the Δ*gem1* single mutant (Figure 1B). The Δ*gem1* cells were resistant to another azole, ketoconazole, and the resistance was dependent on other ERMES components (Supplemental Figure S1). These results indicate that the effect of Gem1 on azole susceptibility requires a functional ERMES complex.

**Figure 2:**
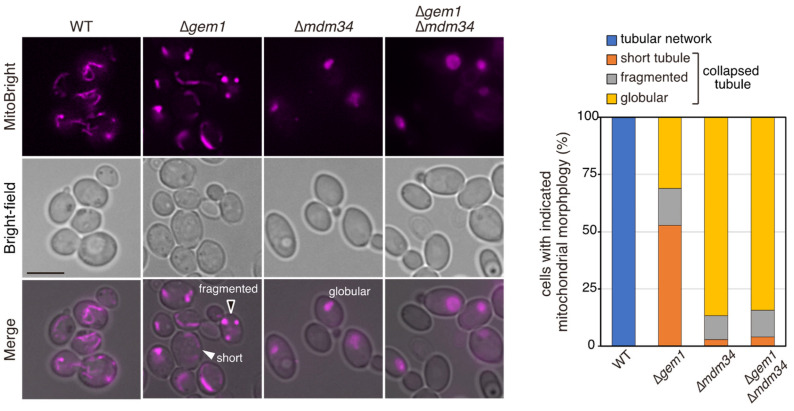
Mitochondrial morphology in Δ*gem1* and Δ*mdm34* cells. Exponentially growing cells were incubated at 37 °C in minimum SD medium, stained with MitoBright LT Red, and observed by fluorescence microscopy. The percentage of cells with the indicated mitochondrial morphology for each strain is shown in the bar graph (*n* = 200). Scale bar, 5 µm.

To evaluate the effect of Gem1 and Mdm34 on mitochondrial morphology in *C*. *glabrata*, the mitochondria-specific dye MitoBright LT Red was used (Figure 2). Under the selected conditions, the wild-type cells displayed a branched tubular mitochondrial network. However, in the absence of *GEM1* mitochondrial morphology, the Δ*gem1* cells displayed shortened or collapsed tubular mitochondrial networks. In Δ*mdm34* cell mitochondria, morphological abnormalities were even clearer, and these cells displayed mostly globular mitochondrial morphology. Furthermore, Δ*mdm34* cells displaying no mitochondria-specific signals upon staining were often observed. Similar mitochondrial morphological abnormalities have been reported in Δ*gem1* and Δ*mdm34* cells of *S. cerevisiae* [21,27]. These results suggested that the ERMES complex is required for normal mitochondrial morphology in *C*. *glabrata.* Furthermore, mitochondrial morphological abnormalities in the Δ*gem1*Δ*mdm34* double deletion mutant were found to be more similar to Δ*mdm34* cells than to Δ*gem1* cells, indicating that the ERMES complex might still be partially functional in Δ*gem1* cells but not in the absence of its core components, such as Mdm34.

### 3.2. The Azole Resistance Phenotype of Δgem1 Cells Depends on Pdr1-Mediated CDR1 Upregulation

**Figure 3:**
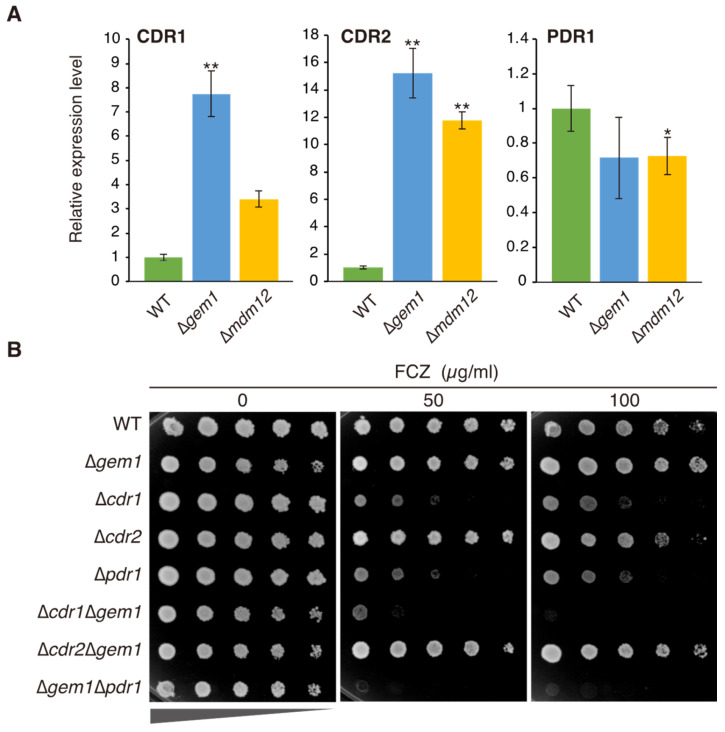
Involvement of *CDR* genes in fluconazole resistance in Δ*gem1* cells. (**A**) Quantitative RT-PCR for *CDR1*, *CDR2*, and *PDR1* in wild-type, Δ*gem1*, and Δ*mdm12* cells. The cells were exponentially incubated at 37 °C in minimal SD medium. Data were normalized to the corresponding levels of the housekeeping transcript (*TEF1*), and the expression levels of WT were set at a relative expression of 1. Values are presented as the mean ± standard error of 3 independent experiments. (**B**) Growth of PDR gene mutants in fluconazole medium. Indicated cells were spotted in 4-fold serial dilutions, as indicated by triangles, on agar plates of minimal SD medium containing fluconazole at the indicated concentrations. The cells were incubated at 37 °C in minimal SD medium for 3 days. *p-*values of the *t*-test vs. wild-type are as follows: * *p* < 0.05, ** *p* < 0.01.

In *C*. *glabrata*, petite mutants with mitochondrial DNA (mtDNA) deficiency display increased resistance to azoles, which is associated with the upregulation of drug efflux pumps of the ABC superfamily [14]. Since abnormal mitochondrial morphology was observed in Δ*gem1* cells, we investigated whether the azole resistance phenotype exhibited by Δ*gem1* cells was mediated by the upregulation of azole drug efflux pumps. The mRNA levels of *CDR1* and *CDR2*, which encode the major azole resistance drug efflux pumps, were assessed using quantitative RT-PCR in wild-type, Δ*gem1,* and Δ*mdm12* cells cultivated in an SD medium. Interestingly, the expression of *CDR1* was upregulated in both Δ*gem1* and Δ*mdm12* cells but was approximately 4-fold higher in Δ*gem1* cells than in Δ*mdm12* cells (Figure 3A). The expression of *CDR2* was also upregulated in both Δ*gem1* and Δ*mdm12* cells but was slightly higher in Δ*gem1* cells than in Δ*mdm12* cells. In addition, the mRNA levels of the transcription factor encoding *PDR1* were also evaluated and found to be virtually unaffected by the deletion of *GEM1* or *MDM12*. These results suggest that in Δ*gem1* cells, the upregulation of *CDR1* or *CDR2* is not caused by increased transcription of *PDR1* but likely through the activation of the encoding transcription factor, even in the absence of azole drugs.

Furthermore, to assess the eventual impact of the PDR genes on the azole resistance phenotype exhibited by Δ*gem1* cells, double deletion mutants devoid of *GEM1* and each PDR gene were constructed. Under the selected conditions, the single deletion of *CDR1* and *PDR1*, but not of *CDR2*, led to fluconazole susceptibility. However, the azole resistance of Δ*gem1* cells was suppressed by the deletion of *CDR1* or *PDR1*, indicating that the azole-resistant phenotype in Δ*gem1* cells is dependent on the PDR1-mediated upregulation of *CDR1*. Furthermore, the deletion of *GEM1* led to increased azole sensitivity in Δ*cdr1* or Δ*pdr1* cells.

### 3.3. The GTPase Activity of Gem1 Is Required for Its Interaction with the ERMES Complex and Azole Susceptibility

**Figure 4:**
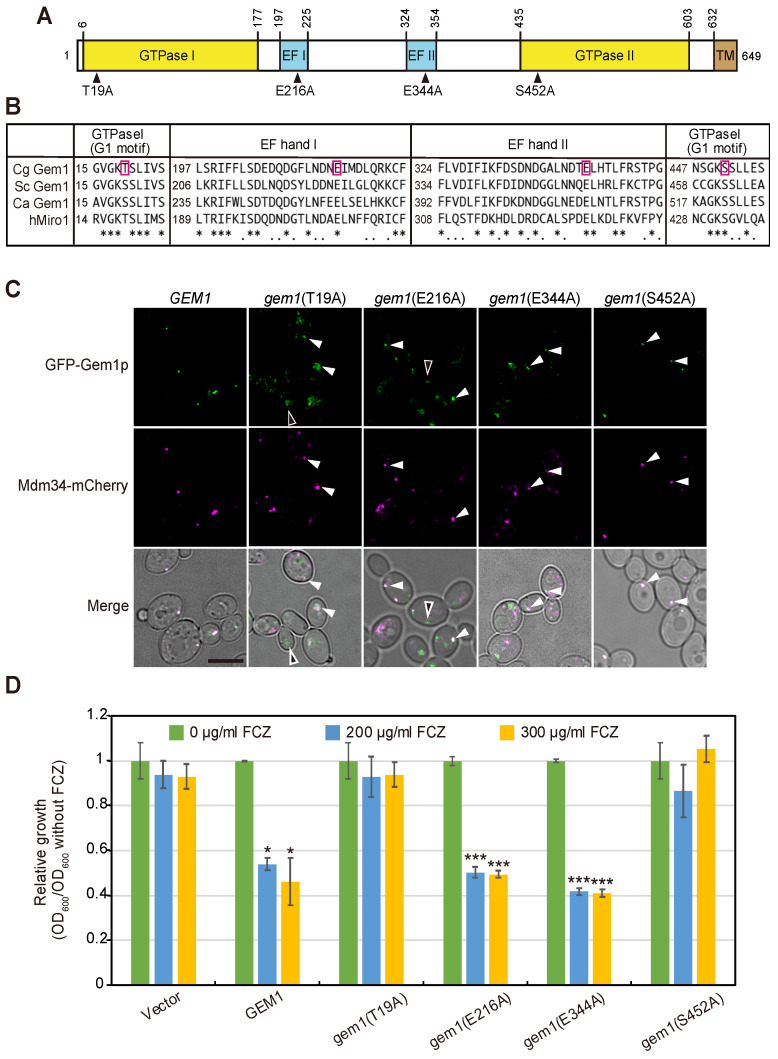
Domain structure of Gem1 and the effects of domain mutations on azole susceptibility. (**A**) Schematic diagram of Gem1, containing 2 putative GTPase domains (GTPase I and II), 2 EF-hand motifs (EF-hand I and II), and a transmembrane domain (TM). The positions indicated by arrows indicate the amino acid substitution sites. (**B**) Sequence alignment of the predicted GTP-binding domain (G1 motif) and EF-hand motifs of Gem1 from *C. glabrata, S. cerevisiae, C. albicans,* and their human ortholog, MIRO1 (hMiro1). The characters in the red frame indicate the mutated residues in this study. (**C**) Localization of mutant Gem1 harboring a mutation in the GTPase domain or Ca^2+^- binding domain. Δ*gem1* cells expressing the indicated GFP-tagged mutant Gem1 under the control of the *GEM1* promoter and Mdm34-mCherry were incubated at 30 °C in SC-Ura medium and observed by confocal microscopy. White arrowheads indicate colocalization between Gem1 and Mdm34. Black arrowheads indicate that Gem1 was not localized in ERMES. Scale bar, 5 µm. (**D**) Δ*gem1* cells, which were transformed with pGRB2.0, pGRB-GFP-*GEM1*, pGRB-GFP-gem1(T19A), pGRB-GFP-gem1 (E216A), pGRB-GFP-gem1 (E344A), or pGRB-GFP-gem1(S452A), were incubated at 30 °C in SC-Ura medium for 1 day and the optical density (OD) at 600 nm was measured. Relative growth was determined by normalizing to OD_600_ without fluconazole. Error bars represent standard errors of 3 independent experiments. *p-*values of the *t*-test against the without FCZ vs. follows: * *p* < 0.05, ** *p* < 0.01, *** *p* < 0.001.

*C. glabrata* Gem1 was predicted to contain two GTPase domains, two calcium-binding EF-hands, and a transmembrane domain (Figure 4A). In *S*. *cerevisiae*, GTP hydrolysis by Gem1 is decreased by the amino acid substitution of serine S19 of the first GTPase domain or S462 of the second domain; and by the amino acid substitution of glutamic acid E225 of the first EF-hand domain or E354 of the second EF-hand domain [42]. To investigate whether these Gem1 domains are required in *C*. *glabrata*, site-directed mutagenesis was used to construct T19A, E216A, E344A, or S452A *gem1* point mutants (Figure 4B). These mutated *gem1* genes were fused with a green fluorescent protein (*GFP*)-encoding gene and introduced into a *CEN*/*ARS*-based low-copy plasmid. The plasmid expressing GFP-Gem1 restored the growth defect of Δ*gem1* cells, suggesting that this fusion protein is functional (Supplemental Figure S2). To evaluate whether the constructed Gem1 mutated proteins interacted with the core components of the ERMES complex, we observed, by confocal microscopy, their intracellular localization in cells expressing mCherry-tagged Mdm34, used as an ERMES marker. The wild-type GFP-Gem1 always co-localizes to the foci containing Mdm34-mCherry, consistent with their co-existence as subunits of the ERMES complex (Figure 4C). GFP-Gem1(S452A) showed stable dot-like colocalization with Mdm34-mCherry, suggesting that the S452A mutation did not affect the Gem1-ERMES interaction (Figure 4C). The fluorescence of GFP-Gem1 (E344A) was detected predominantly in the region where Mdm34 was localized. In contrast, GFP-Gem1(T19A) and GFP-Gem1 (E216A) were not always co-localized with Mdm34-mCherry (Figure 4C, black arrowhead), suggesting that these mutations hamper, but do not fully prevent, the interaction between Gem1 and the ERMES complex.

Next, we examined the effect of each mutation on the azole resistance in Δ*gem1* cells. As expected, the transformation of *GEM1* into Δ*gem1* cells complemented the azole-susceptible phenotype (Figure 4D). Similarly, the expression of *gem1* (E216A) and *gem1* (A344A), which are *GEM1-bearing* mutations in the EF-hand motifs, restored fluconazole susceptibility in Δ*gem1* cells (Figure 4D). In contrast, the expression of *gem1* (T19A) and *gem1* (S452A), which are *GEM1-bearing* mutations in the GTPase domains, did not complement the azole resistance phenotype exhibited by Δ*gem1* cells. Taken together, these results suggest that the GTPase activity of Gem1 is required for ERMES-mediated azole susceptibility in *C. glabrata*. 

### 3.4. Gem1-Dependent Mitochondrial ROS Concentration Affects Azole Susceptibility

**Figure 5:**
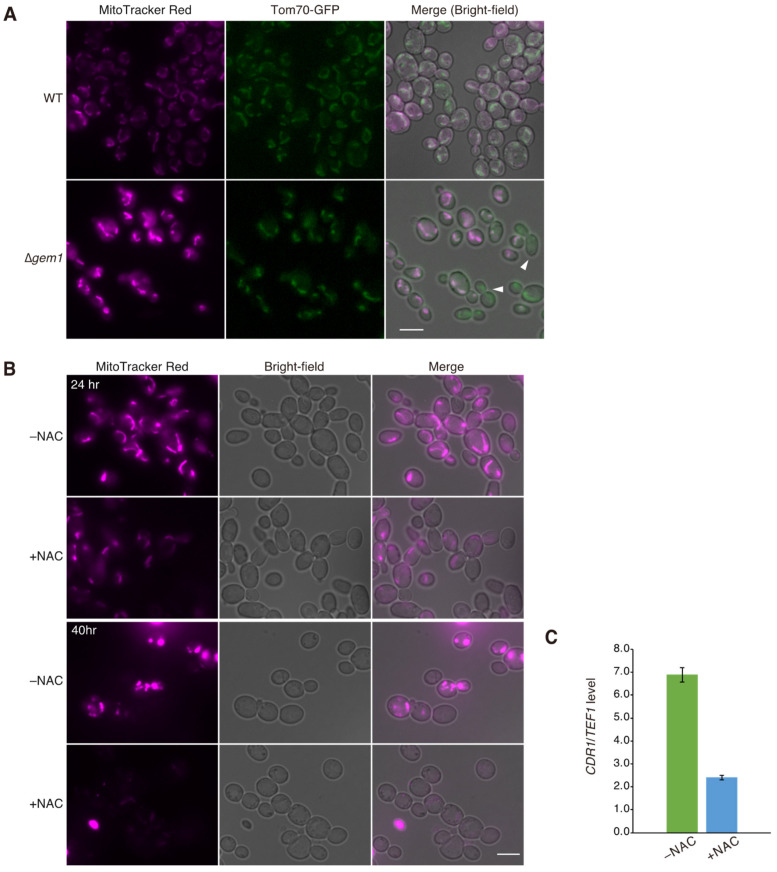
Mitochondrial ROS production in Δ*gem1* cells. (**A**) Detection of intracellular mitochondrial ROS (mtROS). Wild-type and Δ*gem1* cells expressing Tom70-GFP were cultivated in minimal SD medium until the exponential growth phase at 30 °C. The cells were stained with MitoTracker Red CM-H_2_XROS, washed, and observed using fluorescence microscopy. Tom70 was used as a mitochondrial marker. Arrowheads indicate cells in which mitochondria were present but not stained by MitoTracker Red CM-H_2_XROS. Δ*gem1* cells contained approximately 29% cells (*n* = 310) that were not stained by MitoTracker or weakly stained. Scale bar, 5 µm. (**B**) Effect of N-acetylcysteine (NAC) on ROS production in Δ*gem1* cells. Δ*gem1* cells were grown in SD medium with (+NAC) or without 10 mM NAC (–NAC) at 30 °C for 24 or 40 h and then stained with MitoTracker Red. (**C**) Effect of NAC on *CDR1* expression level in Δ*gem1* cells. Δ*gem1* cells were exponentially incubated in SD medium with (+NAC) or without 10 mM NAC (–NAC) at 30 °C for 40 h. *CDR1* expression level was analyzed by quantitative RT-PCR and normalized to the corresponding levels of the housekeeping transcript, *TEF1*. Error bars represent standard errors of 3 independent experiments. The *p*-value of the *t*-test is less than 0.001.

Mitochondria are the main generators of reactive oxide species (ROS). ROS are toxic to cells but are also important signaling molecules. Therefore, we investigated whether mitochondrial morphological abnormalities induced by the deletion of *GEM1* affected ROS concentration in the mitochondria. To detect mitochondrial ROS (mtROS), we used MitoTracker Red CM-H_2_XROS, a reduced dye that fluoresces upon oxidation. Interestingly, stronger fluorescent signals are observed in Δ*gem1* cells than in wild-type cells (Figure 5A). We also noticed that some *Δgem1* cells were not stained with MitoTracker Red CM-H_2_XROS (Figure 5A). To confirm that the stronger signals in Δ*gem1* cells were due to increased mtROS production, we treated Δ*gem1* cells with N-acetylcysteine (NAC), an antioxidant known to reduce ROS generation. Treatment with NAC for 24 or 40 h resulted in a significant reduction of fluorescent signals in Δ*gem1* cells compared to untreated cells (Figure 5B). These results suggest that the absence of *GEM1* contributes to increasing ROS concentrations, likely as a consequence of mitochondrial dysfunction.

To investigate whether ROS production in Δ*gem1* cells is responsible for the elevated expression of *CDR1,* we examined mRNA levels of *CDR1* using quantitative RT-PCR. We found that treatment with NAC, which reduces ROS production in Δ*gem1* cells, clearly decreased the expression of *CDR1* compared to untreated cells (Figure 5C). This result suggested that ROS production in Δ*gem1* cells plays a role in the increased expression of *CDR1*.

## 4. Discussion

Mitochondrial dysfunction is an important factor underlying azole resistance in *C. glabrata* [11,14,15]. Recent studies have shown that azole drugs accumulate in mitochondria, at least upon contact with *C. albicans* cells [43]. However, the molecular mechanisms connecting the azole mode of action, mitochondrial activity and/or dysfunction, and azole resistance are unclear. In a study on *A. fumigatus*, mutants with defects in mitochondrial fission were reported to be azole-resistant [18]; therefore, we focused on the association between mitochondrial dynamics and azole resistance. As the ERMES plays an important role in mitochondrial fission [33], the impact of each subunit on azole resistance was evaluated. Interestingly, only a single deletion of *GEM1* affected azole resistance; however, as azole resistance was suppressed in mutants with double deletions of *GEM1* and additional ERMES components (Figure 1B), the effect of *GEM1*-deletion on azole resistance depended on the remaining components. *Δgem1* cells showed abnormal mitochondrial morphology, increased mtROS, and upregulation of the drug efflux pump encoded by *CDR1*.

Mitochondria are dynamic organelles, and their continuous fission and fusion are necessary for the maintenance of mitochondrial function. The fission site of mitochondria is wrapped around by ER tubules [44], where the ERMES complex localizes [33]. At the fission site of mitochondria, the ERMES complex localizes adjacent to replicating mtDNA and is involved in the distribution of newly replicated mtDNA to the dividing mitochondria [33]. After mitochondrial fission, one of the dividing mitochondria is detached from the ER, a process that requires Gem1 in *S. cerevisiae* [33]. In particular, deletion mutants of the ERMES core complex display defects in the segregation of mtDNA into daughter cells, resulting in the loss of mitochondria over several generations of growth in *S*. *cerevisiae* [27,29,30,45]. Similarly, in *C*. *glabrata*, some cells losing mitochondria were observed in Δ*mdm34* (Figure 2A), suggesting that the deletion of *MDM34* mainly induces abnormal mtDNA distribution. The deletion of the ERMES core complex did not result in azole resistance (Figure 1B); defects in mtDNA distribution might not be associated with azole resistance. In contrast, the deletion of *GEM1* conferred resistance to azoles (Figure 1B). *C. glabrata* mitochondria in *Δgem1* cells had a morphology similar to that observed in *S. cerevisiae Δgem1* cells (Figure 2A), suggesting that CgGem1 also contributes to the disruption of contact between the ER and mitochondria after mitochondrial fission. We found that azole resistance in Δ*gem1* cells was suppressed by the deletion of the ERMES core complex, Mdm10, Mdm12, or Mdm34 (Figure 1B). Deletion of any four proteins of the ERMES core complex results in the impaired formation of the ERMES complex and instability of ER-mitochondrial contact [21]. Therefore, the ERMES complex-mediated contact between the ER and mitochondria is a crucial factor in the acquisition of azole resistance in *C*. *glabrata*.

Gem1 contains two GTPase domains and two Ca^2+^-binding EF-hand domains. In *S*. *cerevisiae*, mutations leading to decreased GTPase activity, Gem1(S19N) or Gem1(S462N), impair the maintenance of mitochondrial morphology and dissociation of ERMES from the fission site [33]. Significantly, we showed that similar amino acid substitutions in the GTPase domains of *C*. *glabrata* Gem1 were also sufficient to confer azole resistance (Figure 4D). The fact that single mutations in *GEM1* are sufficient to increase azole resistance suggests that *GEM1* mutations may confer azole resistance in *C. glabrata* clinical isolates.

In Δ*gem1* cells, the mRNA expression of the efflux pump-encoding genes *CDR1* and *CDR2* was found to be upregulated (Figure 3A). Furthermore, the deletion of *CDR1* or *PDR1* suppressed the azole-resistant phenotype of Δ*gem1* cells. (Figure 3B). These results indicate that the deletion of *GEM1* leads to azole resistance by upregulating the expression of *CDR1* via Pdr1 activation. The deletion of *GEM* increased the sensitivity of Δ*cdr1* or Δ*pdr*1 to azole. It supposes that Pdr1 and Cdr1 are required for mtROS processing and that excess mtROS induced by *GEM1* deletion reduces cell proliferation. Consequently, the deletion of *GEM1* in Δ*pdr1* or Δ*cdr1* cells indicated an additive effect on fluconazole sensitivity. Double deletion of *CDR2* and *GEM1* indicated greater azole resistance than deletion of *CDR2* alone; thus, *CDR2* seems to have little effect on the mtROS processing. We also found that mtROS production is enhanced in Δ*gem1* cells (Figure 5A). The treatment with NAC, an antioxidant, reduced the mtROS production in Δ*gem1* cells (Figure 5B) and inhibited the upregulating of the expression of *CDR1* (Figure 5C), implying that mtROS may be involved in the activation of Pdr1. Therefore, we propose that in Δ*gem1* cells, increased mtROS production caused by mitochondrial dysfunction directly or indirectly activates Pdr1 and upregulates the drug efflux pump Cdr1, leading to azole resistance. An increase in intracellular ROS levels causes oxidative stress. In *Kluyveromyces lactis*, *KI*Upc2, whose homolog activates Pdr1 in response to fluconazole in *C*. *glabrata* [46], has been reported to be involved in the oxidative stress response [47]. In *C*. *glabrata*, Upc2 may activate Pdr1 in response to oxidative stress. However, it is not known whether oxidative stress or mtROS regulates the activation of Pdr1, and further analysis of Δ*gem1* cells is needed.

As excess ROS is toxic to cells, cells must remove excess ROS and maintain a low concentration of ROS. We observed some cells that were not stained or very weakly stained with MitoTracker Red CM-H_2_XROS in Δ*gem1* cells (Figure 5A). No such cells were observed in the wild-type cells. The cells with reduced mtROS in Δ*gem1* cells imply that the antioxidant system, such as scavenging excess mtROS, may work actively in Δ*gem1* cells. Mitochondrial fission has been reported to be involved in the release of cytochrome C from mitochondria, which acts as a scavenger of ROS in the cytosol [48]. Analysis of Δ*gem1* cells may reveal the mechanism by which excess mtROS is scavenged.

Altogether, the results described herein suggest that Gem1 is required to maintain mitochondrial dynamics and redox state in *C. glabrata*. In the absence of Gem1 activity, caused either by gene deletion or by point mutations in its GTPase domains, the mitochondrial ROS concentration increases, leading to Pdr1-dependent upregulation of the drug efflux pumps Cdr1 and Cdr2, resulting in azole resistance. This knowledge is expected to contribute to the development of much-needed antifungal molecules that target mitochondrial-related azole drug resistance.

## Data Availability

Not applicable.

## Supplementary Materials

The following supporting information can be downloaded at: https://www.mdpi.com/article/10.3390/jof9020240/s1, Figure S1: Growth of strains lacking ERMES components in the presence or absence of ketoconazole; Figure S2: Expression of GFP-GEM1 plasmid in Δ*gem1* cells; Table S1: List of strains; Table S2: List of PCR primers [49,50].
