# Supplementary material for: In Candida glabrata, ERMES Component GEM1 Controls Mitochondrial Morphology, mtROS, and Drug Efflux Pump Expression, Resulting in Azole Susceptibility
Source: J Fungi (Basel). 2023 Feb 10;9(2):240. doi: 10.3390/jof9020240 (PMC9965728; doi:10.3390/jof9020240)
Supplement: Supplementary file 1 [file jof-09-00240-s001.zip › jof-2051761-supplementary.pdf]

Table S1 List of strains

Strain	parent	genotype	Reference
2001HTU		Δ*his3* Δ*trpl* Δ*ura3*	[49]
KUE100		*his3 yku80*::*SAT1* flipper	[50]
Δ*mdm10*	KUE100	*mdm10*::*CgHIS3*	This study
Δ*mdm12*	KUE100	*mdm12*::*CgHIS3*	This study
Δ*mdm34*	KUE100	*mdm34*::*CgHIS3*	This study
Δ*mmm1*	KUE100	*mmm1*::*CgHIS3*	This study
Δ*gem1*	KUE100	*gem1*::*CgHIS3*	This study
Δ*gem1*Δ*mdm10*	KUE10	*gem1*::loxP *mdm10*::*CgHIS3*	This study
Δ*gem1*Δ*mdm12*	KUE100	*gem1*::loxP *mdm12*::*CgHIS3*	This study
Δ*gem1*Δ*mdm34*	KUE100	*gem1*::loxP *mdm34*::*CgHIS3*	This study
Δ*gem1*Δ*mmm1*	KUE100	*gem1*::loxP *mmm1*::*CgHIS3*	This study
Δ*gem1* (V)	2001HTU	*gem1*::loxP, pGRB2.0	This study
Δ*gem1* (GEM1)	2001HTU	*gem1*::loxP *MDM34*::*mCherry-natMX6*, pGRB-GFP-*GEM1*	This study
Δ*gem1* (S19A)	2001HTU	*gem1*::loxP *MDM34*::*mCherry-natMX6*, pGRB-GFP-gem1(T19A)	This study
Δ*gem1* (E216A)	2001HTU	*gem1*::loxP *MDM34*::*mCherry-natMX6*, pGRB-GFP-gem1 (E216A)	This study
Δ*gem1* (E344A)	2001HTU	*gem1*::loxP *MDM34*::*mCherry-natMX6*, pGRB-GFP-gem1 (E344A)	This study
Δ*gem1* (S452A)	2001HTU	*gem1*::loxP *MDM34*::*mCherry-natMX6*, pGRB-GFP-gem1 (S452A)	This study

Table S2 List of PCR primers

Primer	sequence (5'→3')
CDR1-F	ATGCTAACCCAGCTGAATGG
CDR1-R	TCTTTGGAAGCTCGTTGGAC
CDR2-F	CGCCAACCATGTAACAGATG
CDR2-R	TTTGAGCCACAGATCCACAC
PDR1-F	GTGAAGCGGAAGAAATGCTC
PDR1-R	TATTCGTCGAGAGCAAGCTG
GFP-GEM1-F1	cactaaagggaacaaaagctggagctcCCAGATTTGATACTGTACACGTC
GFP-GEM1-R1	ctttactcattctagaTGATAATCCTGTTACAGCCT
GFP-GEM1-F2	aggattatcatctagaATGAGTAAAGGAGAAGAACT
GFP-GEM1-R2	cttttgtcatgtcgacTCTAGATTTGTATAGTTCATCCATGCC
GFP-GEM1-F3	actatacaaatctagagtcgacATGACAAAAGAAACCATCAG
GFP-GEM1-R3	ctcactatagggcgaattgggtaccTATTTATCTTGGTCAAGAAT
pGRB2.1-F1	GTACCCAATTCGCCCTATAGTGA
pGRB2.1-R1	AGGATTATCACTCCAGCTTTTGTTCCCTTTAGTG
MDM34-F1	GAAAAGAGACTCAAAAGAGCG
MDM34-R1	GTTAATTAACCCGGGGATCCGATAATATGGTGGTGGTGGCG
MDM34-F2	GTTTAAACGAGCTCGAATTCGGTATTTTTAAGCGTTATTGCTA
MDM34-R2	CAACGGTCAACAACATCTTC
PFA6A-F	CGGATCCCCGGGTTAATTAAC
pFA6a-R	GAATTCGAGCTCGTTTAAAC
TOM70-F1	CATGATGCTACTGAAGAGTCGG
TOM70-R1	GTTAATTAACCCGGGGATCCGAAATCCTTGTCCACCATTACGAC
TOM70-F2	GTTTAAACGAGCTCGAATTCATAAGCGAGCTCCTAAACCAGAC
TOM70-R2	AAGGCATTAGCCTTCTCAAACG

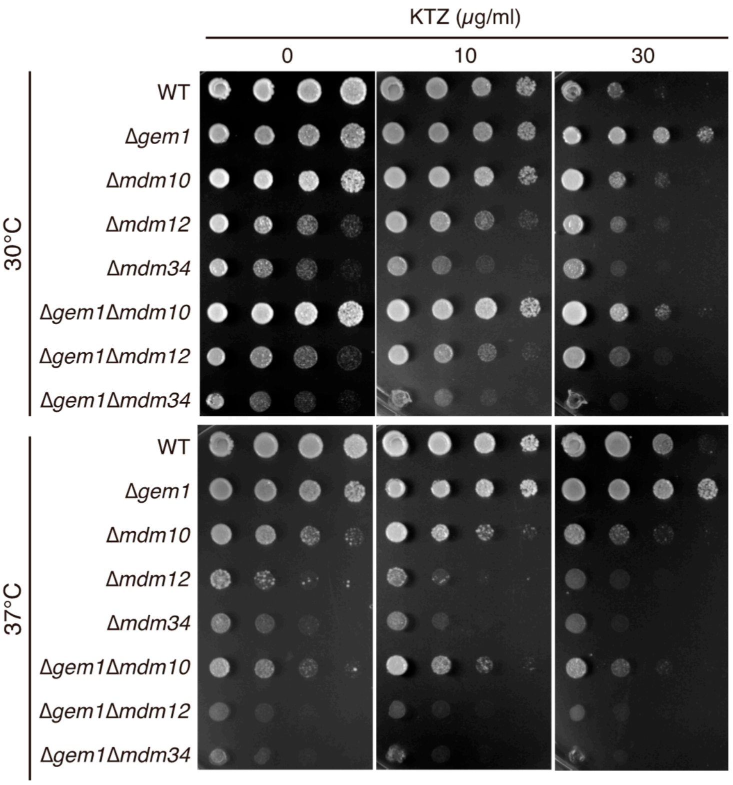

Supplemental figureS1

Growth of strains lacking ERMES components in the presence or absence of ketoconazole. The cells were diluted to OD_{600} of 0.5 in water and spotted in 4-fold serial dilutions (indicated by triangles) on minimal SD medium containing the indicated concentration of ketoconazole (KTZ). Cells were incubated for two days at 30 °C or 37 °C.

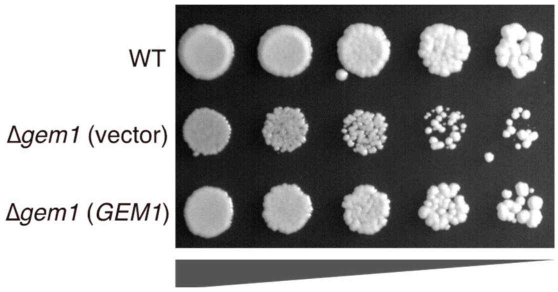

Supplemental figureS2

Expression of GFP-GEM1 plasmid in Δ*gem1* cells. Wild-type cells (KUE100) and Δ*gem1* cells, which were transformed with pGRB2.0 (vector) or pGRB-GFP-*GEM1*(*GEM1*), were spotted in 4-fold serial dilutions, as indicated by triangles, and incubated at 30 °C in SC-Ura medium for 3days.
